# Mendelian randomization analysis reveals fresh fruit intake as a protective factor for urolithiasis

**DOI:** 10.1186/s40246-023-00523-2

**Published:** 2023-10-03

**Authors:** Yiwei Lin, Cheng Zhou, Yuqing Wu, Hong Chen, Liping Xie, Xiangyi Zheng

**Affiliations:** 1 Department of Urology, The First Affiliated Hospital, Zhejiang University School of Medicine, Hangzhou, 310003 Zhejiang China; 2 Department of Urology, The First Affiliated Hospital of Ningbo University, Ningbo, 315010 Zhejiang China

**Keywords:** Food intake, Urolithiasis, Mendelian randomization, Kidney stone disease

## Abstract

**Objective:**

Previous studies have proposed that food intakes are associated with the risk of urolithiasis. Here, we conducted a two-sample Mendelian randomization (MR) study to evaluate the causal effects of different food intakes on urolithiasis.

**Methods:**

Independent genetic variants associated with different food intakes at a genome-wide significant level were selected from summary-level statistics of genome-wide association studies from the UK Biobank. The association of these instrumental variables with urolithiasis was studied in a cohort from FinnGen Consortium.

**Results:**

Among the 15 studied food intake exposures, tea intake (odds ratio [OR] = 0.433, 95% confidence interval [CI] = 0.281–0.667, *p* value = 1.470 × 10^–4^) and fresh fruit intake (OR = 0.358, 95% CI = 0.185–0.694, *p* value = 0.002) were found to significantly reduce the risk of the calculus of kidney and ureter. The association remained consistent in the sensitivity analyses. After adjusting for the effects of vitamin D and vitamin C, fresh fruit intake remained the reverse causal association with the calculus of kidney and ureter.

**Conclusions:**

Genetically proxied fresh fruit intake is causally associated with a reduced risk of the calculus of kidney and ureter.

**Supplementary Information:**

The online version contains supplementary material available at 10.1186/s40246-023-00523-2.

## Introduction

Urolithiasis is a common medical condition characterized by high prevalence, with over 15% of the world’s population afflicted [1]. In the last few years, the incidence and prevalence of urolithiasis kept rising in both males and females, laying a great burden on the healthcare systems [2, 3]. The pathogenesis of renal stones is affected by a variety of environmental factors. Previous publications have indicated that nutritional exposures, i.e., food intakes, are potentially one of the most important factors involved in the increased incidence of urolithiasis [4]. Furthermore, kidney stones are found to be influenced by genetic predisposition, genetic variants, and polygenic involvement are also important factors in urolithiasis [5]. It is now commonly accepted that nutritional factors are crucial in the prevention of nephrolithiasis and its recurrence [6, 7]. However, previous studies on different food intakes’ effects on nephrolithiasis are mostly based on observational studies, which might be affected by the bias of confounding factors and reverse causality. Whether the association of food intakes with urolithiasis is causal has not been established due to the lack of randomized controlled trials (RCTs). Despite being the gold standard of causal inference, the implementation of randomized controlled trials (RCTs) in this particular case is challenging due to feasibility and ethical considerations [8].

Mendelian randomization (MR) is a method that uses single-nucleotide polymorphisms (SNPs) as instrumental variables to proxy certain exposures [9, 10]. As the random assignment of genetic variants happens during conception, resembling the randomization of RCTs, MR can diminish the risk of environmental or self-adopted factors [11]. Furthermore, since the genetic variants used as proxies for the exposures cannot be influenced by the onset and progression of the disease outcomes, MR minimized the bias from reverse causality [10].

Understanding the exact role of food intakes in urolithiasis may provide useful suggestions for effective prevention and treatment. Thus, here we have used a two-sample MR method to systematically evaluate the causal effects of different food intakes on the risk of urolithiasis.

## Methods

### Study design

**Fig. 1:**
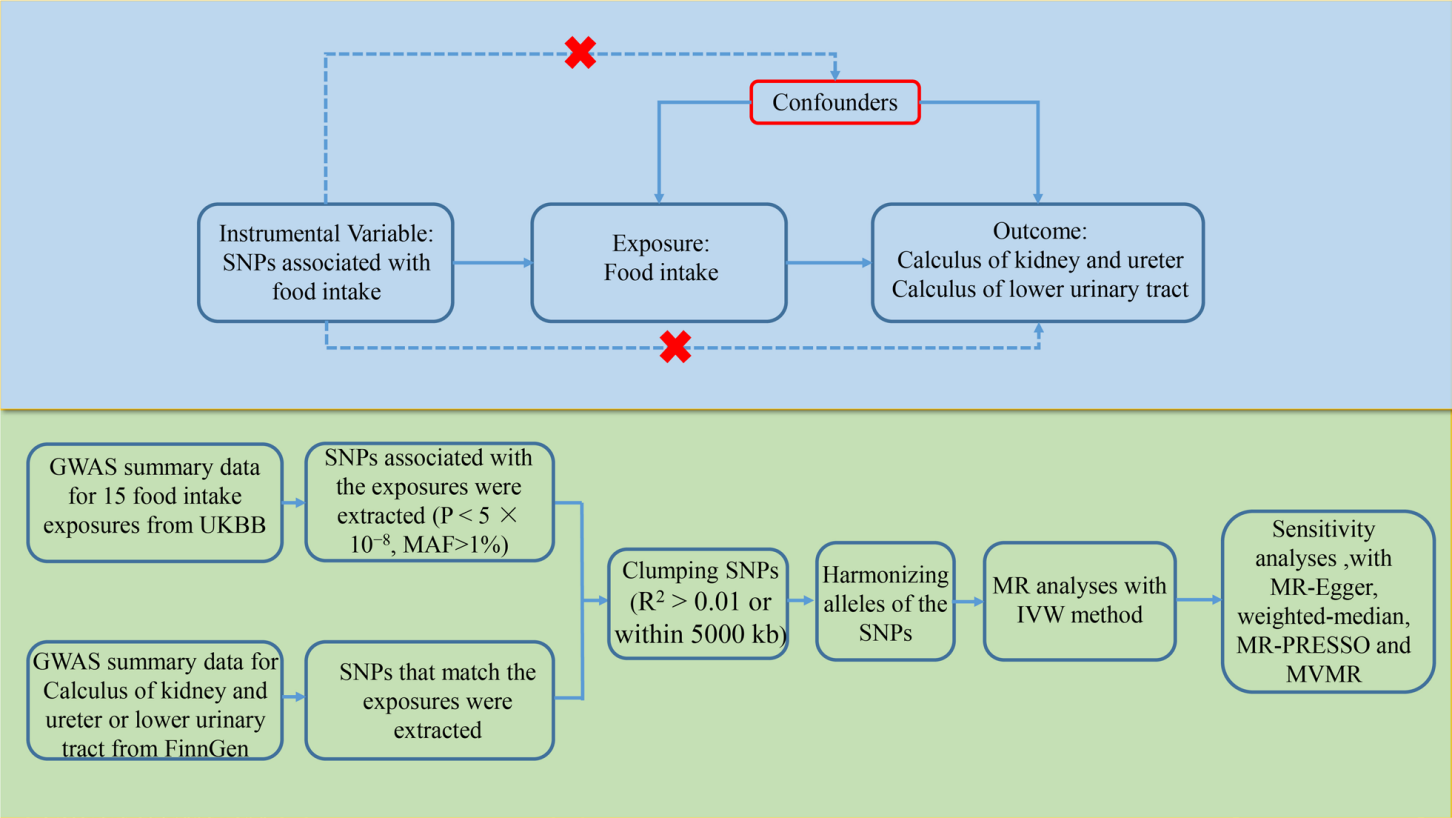
Diagrams showing the three assumptions of MR analyses and the study design overview. MR, Mendelian randomization; SNP, single-nucleotide polymorphism; GWAS, genome-wide association study; UKBB, UK biobank; MAF, minor allele frequency; IVW, inverse-variance weighted; MVMR, multivariable MR

Figure 1 shows the three important assumptions of MR analyses and an overview of the study design. The present MR study included 15 food intake factors. We first assessed the causal effects of different food intakes on the risk of urolithiasis (calculus of kidney and ureter or low urinary tract) by using two-sample MR analyses. We further estimated the causal association between fresh fruit intake and urolithiasis risk after adjusting for the effects of vitamin C and vitamin D with multivariable MR (MVMR). The current study is based on publicly available summary-level statistics from large genome-wide association studies (GWASs) and consortia. Informed consent from the patients was obtained in all the included studies. All included studies were approved by a local review board.

### Data sources

Summary-level statistics of food intake were obtained from UK Biobank (UKBB) [12]. Food intake exposures analyzed in this study included intake of bread, cereal, cheese, coffee, cooked vegetable, dried fruit, fresh fruit, lamb, non-oily fish, oily fish, pork, poultry, processed meat, raw vegetable, and tea. Dietary assessments were performed with a touchscreen questionnaire about the frequency of consumption over the past year of the above-mentioned food, as well as questions on the avoidance of certain foods, changes in dietary habits in the past 5 years, etc. [13].

Summary-level statistics of urolithiasis were obtained from the newest release from the FinnGen consortium (Release 8) [14]. Patients with calculus of kidney and ureter were identified with ICD codes (ICD-10: N20; ICD-9: 592; ICD-8: 592), in total 8,597 cases and 333,128 controls were included in this study. ICD codes were also used to identify patients with calculus of the lower urinary tract (ICD-10: N21; ICD-9: 594; ICD-8: 594), and 1224 cases were identified. Detailed information on the data sources is shown in Table 1.

**Table 1:** Detailed information about data sources of food intakes, circulating levels of vitamin D/vitamin C, and urolithiasis

Traits	GWAS ID*	Consortium	Year	Used SNPs	Sample size	F-statistics
Bread intake	ukb-b-11348	UKBB	2018	33	452,236	18.0272
Cereal intake	ukb-b-15926	UKBB	2018	42	441,640	17.9189
Cheese intake	ukb-b-1489	UKBB	2018	68	451,486	13.6880
Coffee intake	ukb-b-5237	UKBB	2018	44	428,860	31.7542
Cooked vegetable intake	ukb-b-8089	UKBB	2018	17	448,651	17.2863
Dried fruit intake	ukb-b-16576	UKBB	2018	45	421,764	15.7614
Fresh fruit intake	ukb-b-3881	UKBB	2018	58	446,462	19.5377
Lamb intake	ukb-b-14179	UKBB	2018	32	460,006	15.0406
Non-oily fish intake	ukb-b-17627	UKBB	2018	12	460,880	18.6157
Oily fish intake	ukb-b-2209	UKBB	2018	65	460,443	17.8875
Pork intake	ukb-b-5640	UKBB	2018	13	460,162	16.0884
Poultry intake	ukb-b-8006	UKBB	2018	9	461,900	14.4174
Processed meat intake	ukb-b-6324	UKBB	2018	23	461,981	15.1364
Raw vegetable intake	ukb-b-1996	UKBB	2018	21	435,435	14.9826
Tea intake	ukb-b-6066	UKBB	2018	50	447,485	27.1543
Vitamin D level	ebi-a-GCST005367	NA	2018	11	79,366	NA
Vitamin C level	met-a-348	NA	2014	14	2,085	NA
Calculus of kidney and ureter	N14_CALCUKIDUR	FinnGen	2022	NA	341,725^**†**^	NA
Calculus of lower urinary tract	N14_CALCULOWER	FinnGen	2022	NA	334,352^**†**^	NA

^*^Summary statistics of food intakes, vitamin D and C levels can be found on https://gwas.mrcieu.ac.uk/. Summary statistics for urolithiasis can be found on https://r8.finngen.fi/

^†^Calculus of kidney and ureter: 8,597 cases and 333,128 controls; Calculus of lower urinary tract: 1224 cases and 333,128 controls

### Genetic instrument selection

SNPs that were associated with food intakes were extracted with a genome-wide significance threshold (*p* < 5 × 10^−8^). SNPs in linkage disequilibrium were identified and excluded with LD clumping method (*R*^2^ > 0.01 or within 5000 kilobases distance). The proportion of explained trait variance (R^2^) and F-statistics of the IVs were calculated as described in previous publications [15].

### Statistical analysis

For the univariable two-sample MR analyses, the inverse-variance weighted (IVW) method was used as the main analysis model [16]. A random effect IVW method was used when more than three instrumental variables were available; otherwise, a fixed effect IVW method was used. Several other MR methods were employed as sensitivity analyses, including MR-Egger, weighted median, and MR-PRESSO methods [17–19]. MR-Egger regression is a method that can detect and correct for potential horizontal pleiotropy (*p* for intercept < 0.05). The weighted median method can give consistent causal estimates when up to 50% of all used IVs were invalid. MR-PRESSO method can effectively identify outliers of the IVs and give causal estimates after excluding the outliers, thus minimizing the bias from horizontal pleiotropy. Cochrane’s Q-value was calculated to estimate the heterogeneity among the IVs. MVMR is a method that gives direct causal estimates of exposures’ effects on the outcome after adjusting for the effects of other exposures [20]. We have used MVMR in this study to estimate the causal effects of fresh fruit intake on urolithiasis after adjusting for circulating vitamin C and vitamin D levels. To minimize the risk of reverse causality that individuals with urolithiasis may modify their food intakes, we further performed reverse MR analyses to test the causal effects of calculus of kidney and ureter on the food intakes. Statistical power of the MR analyses were calculated with an online tool mRnd (https://shiny.cnsgenomics.com/mRnd/) [21]. HyPrColoc was further used to identify potential colocalization between the exposures and outcomes [22].

All statistical analyses were two-sided. For the main analyses, a *p* value less than 0.003 was considered statistically significant (0.05/15, Bonferroni adjustment for multiple testing). A *p* value between 0.003 and 0.05 was considered suggestively significant. All statistical analyses were performed with “TwoSampleMR” (0.5.5), “Mendelian randomization” (0.5.0), and “MVMR” packages [20, 23, 24].

## Results

### Food intakes and urolithiasis

In total, 15 exposures had enough IVs and were included in this study. The number of SNPs used as IVs for the exposures is shown in Table 1. The F-statistics of all exposures were all higher than 10, indicating a low risk of weak instrument bias.

**Fig. 2:**
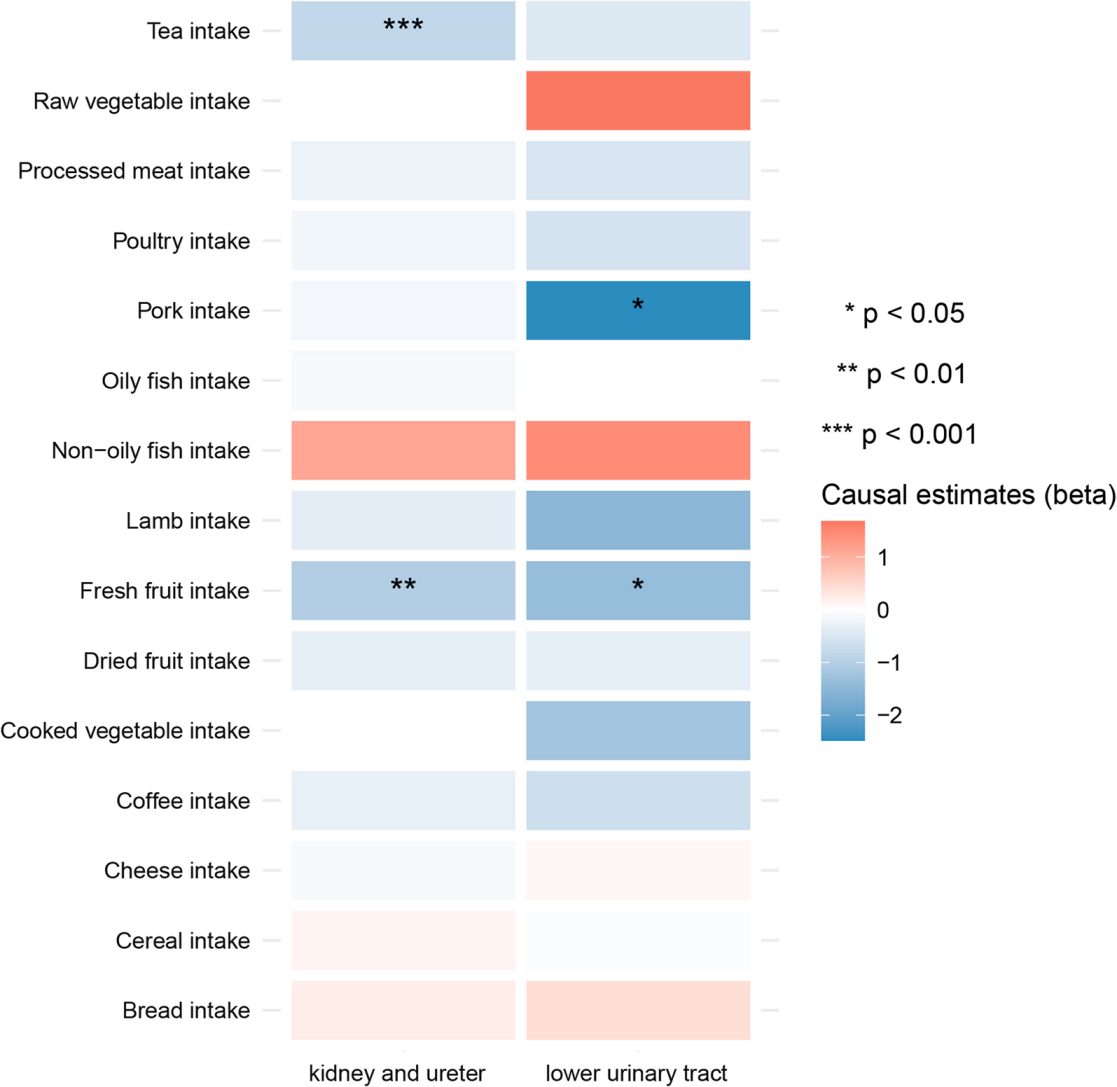
Heatmap showing the causal effects of different food intake on the risk of calculus of kidney and ureter, or calculus of lower urinary tract by using IVW method. IVW: inverse-variance weighted

We first assessed the causal effects of all individual food intake exposure on urolithiasis. Consistent with previous publications, tea intake was found to have a significant protective effect on the risk of calculus of kidney and ureter (odds ratio [OR] = 0.433, 95% confidence interval [CI] = 0.281–0.667, *p* value = 1.470 × 10^–4^) (Fig. 2, Additional file 1: Table S1) [25]. However, the causal effect disappeared when it comes to calculus of lower urinary tract (OR = 0.512, 95% CI = 0.179–1.460, *p* value = 0.330) (Fig. 2, Additional file 1: Table S2). The statistical power for the causal estimates of tea intake on calculus of kidney and ureter was 63% with an OR of 0.433. Fresh fruit intake was also found to significantly reduce the risk of calculus of kidney and ureter (OR = 0.358, 95% CI = 0.185—0.694, *p* value = 0.002), and the causal association retained with calculus of lower urinary tract (OR = 0.247, 95% CI = 0.069–0.889, *p* value = 0.032) (Fig. 2, Additional file 1: Table S1, S2). The statistical power was 67% to detect the effect of fresh food intake on calculus of kidney and ureter with an OR of 0.358, while the power was only 19% for the effect of fresh food intake on calculus of lower urinary tract with an OR of 0.247. We performed colocalization analyses between fresh fruit intake and calculus of kidney and ureter/calculus of lower urinary tract with HyPrColoc; however, no colocalization was identified. No causal correlation was found between dried fruit intake and either kidney stone or lower urinary tract stone (Fig. 2). Pork intake was also found to have a suggestively significant protective effect on the risk of calculus of lower urinary tract, but not kidney and ureter (Fig. 2).

### Sensitivity analyses

**Fig. 3:**
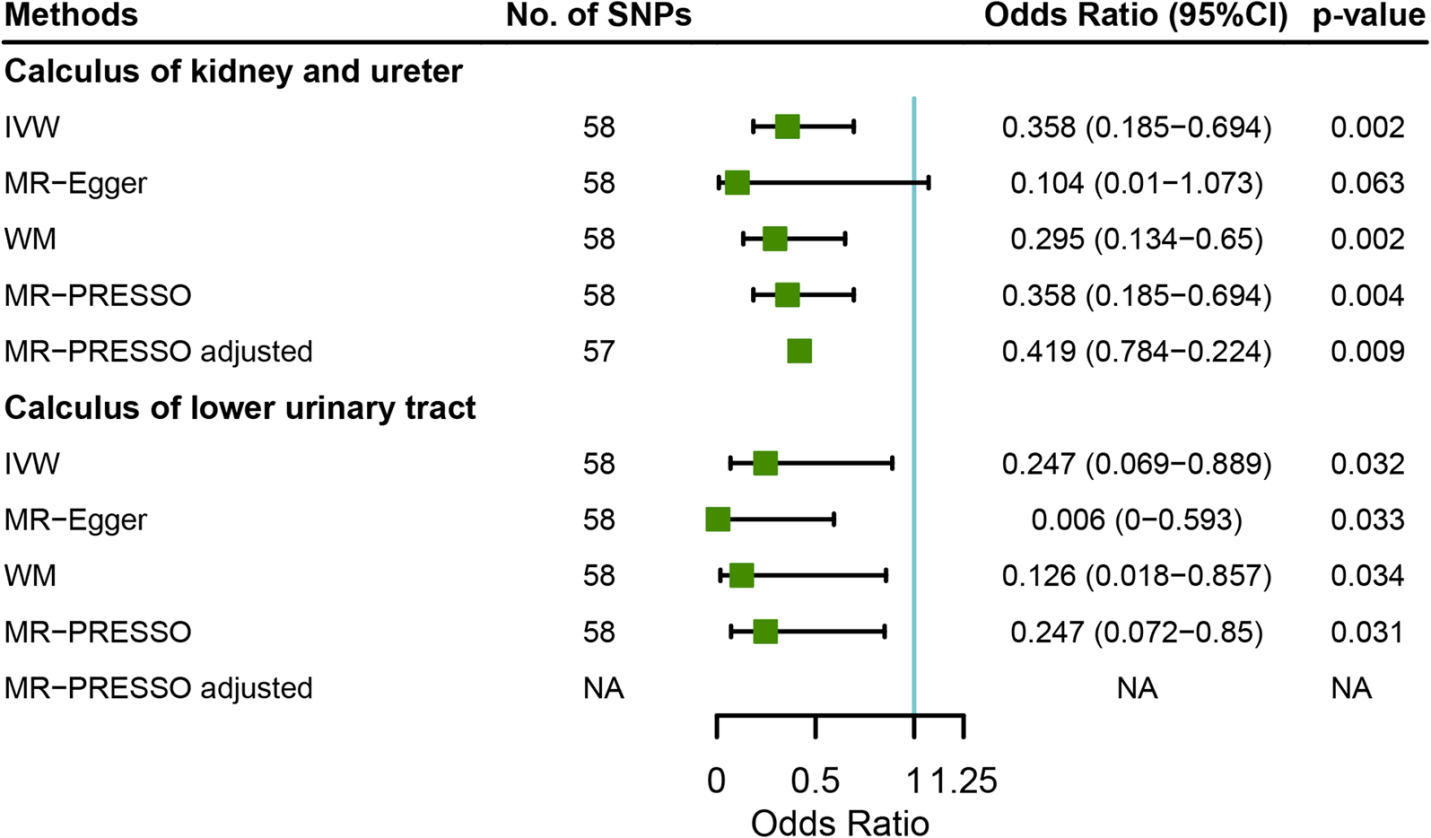
Forest plot showing the causal associations between fresh fruit intake and calculus of kidney and ureter or lower urinary tract by using IVW, MR-Egger, WM, or MR-PRESSO methods. SNP, single-nucleotide polymorphism; IVW, inverse-variance weighted; MR, Mendelian randomization; WM, weighted median

To test the robustness of the results, we further performed several sensitivity analyses. The causal effect of fresh fruit intake on urolithiasis was retained in all sensitivity analyses except in the association with calculus of kidney and ureter by using the MR-Egger method (OR = 0.104, 95% CI = 0.010–1.073, *p* value = 0.063) (Fig. 3, Additional file 1: Tables S3, S4, S5, S6). One outlier was identified by MR-PRESSO method, and excluding the outlier did not influence the causal association (Fig. 3). No horizontal pleiotropy was identified in the analyses with MR-Egger method (Additional file 1: Tables S7, S8). Heterogeneity of the MR analyses are presented in Additional file 1: Table S5 and S11. Significant heterogeneity (*p* value for Cochrane’s Q-value < 0.05) was identified in the MR analyses of several food intake exposures on urolithiasis (Additional file 1: Table S9, S10). We have used a random effect model IVW method in these analyses which reduced the risk of bias from horizontal pleiotropy. SNPs used as instrumental variables for all the exposures, their associations with the exposures and the outcomes are presented in Additional file 1: Table S11 and S12.

### Multivariable MR and reverse MR

**Fig. 4:**
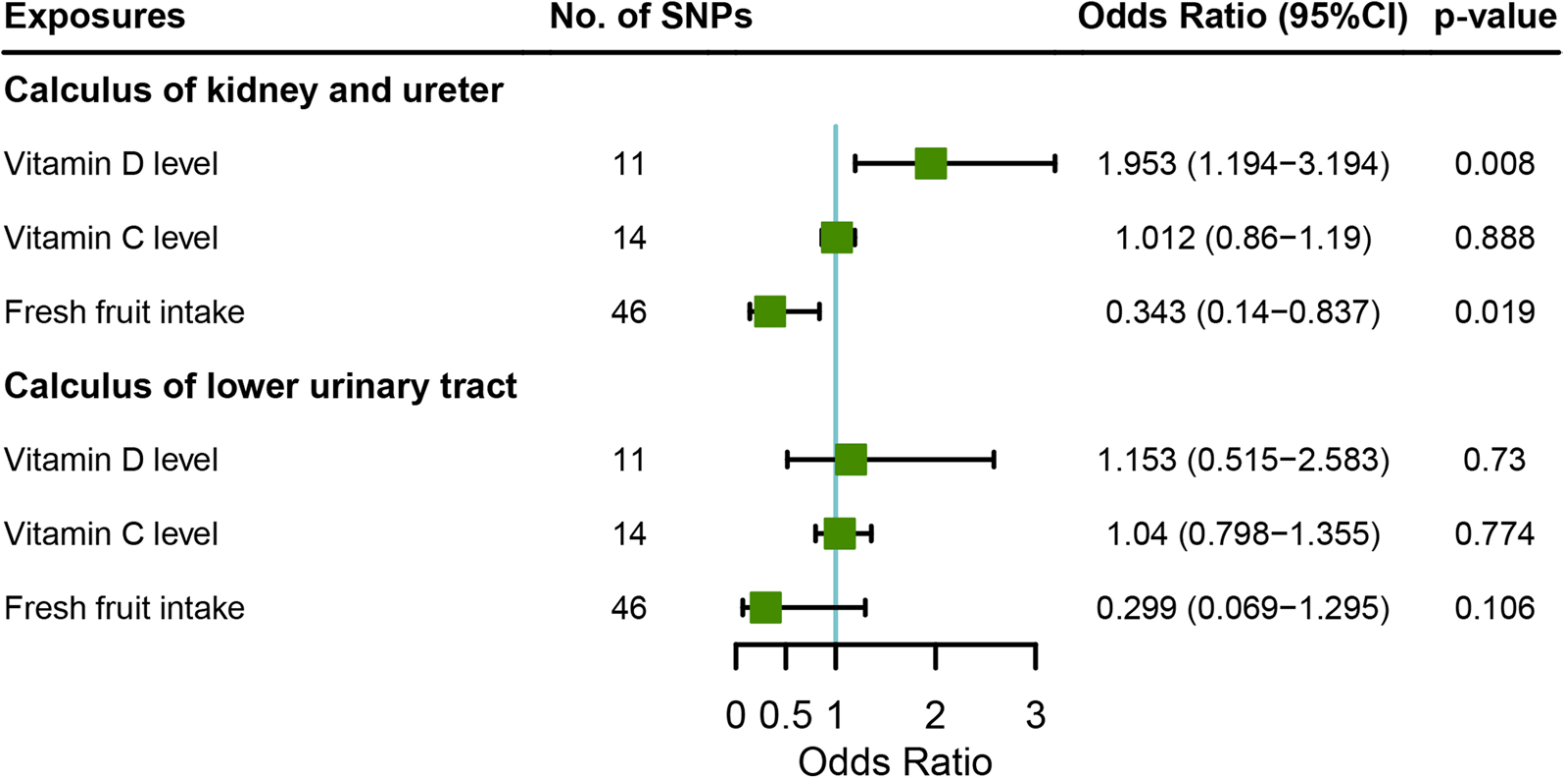
Adjusted causal effects of vitamin D level, vitamin C level, and fresh fruit intake on the risk of calculus of kidney and ureter, or lower urinary tract after adjusting for the effects of other two exposures by MVMR analyses. SNP, single-nucleotide polymorphism; MVMR, Multivariable Mendelian Randomization

To examine if the protective role of fresh fruit intake is mediated by the increased level of circulating vitamin C or vitamin D, we performed a MVMR analysis to adjust for the effects of the circulating vitamin levels. Higher vitamin D level was found to significantly increase the risk of the calculus of kidney and ureter (OR = 1.953, 95% CI = 1.194–3.194, *p* value = 0.008), but not calculus of lower urinary tract (OR = 1.156, 95% CI = 0.516–2.590, *p* value = 0.725) (Fig. 4). Vitamin C level is not associated with urolithiasis in the MVMR analyses (Fig. 4). Fresh fruit intake remained to have a significant protective effect on calculus of kidney and ureter remained significant after adjusting for vitamin C and vitamin D (OR = 0.343, 95% CI = 0.14–0.837, *p* value = 0.019), but not on calculus of lower urinary tract (OR = 0.299, 95% CI = 0.069–1.295, *p* value = 0.106).

To test the possibility of reverse causality that individual with urolithiasis may change their food choices, we calculated the causal effects of calculus of kidney and ureter on the 15 food intakes. However, we observed no significant causal effects with the IVW method (Additional file 1: Table S13).

## Discussion

In this study, we have used two-sample MR to evaluate the causal relationship between a variety of food intakes and the risk of urolithiasis. In line with previous publications, our study supported a protective role of tea intake in calculus of kidney and ureter, but not in calculus of lower urinary tract [25, 26]. Furthermore, we have identified fresh fruit intake, instead of dry fruit intake, as a reverse causal factor for urolithiasis. After adjusting for the effect of vitamin C and vitamin D, fresh fruit intake remained to have a protective effect on calculus of kidney and ureter.

It is now well-accepted that dietary factors are crucial in the prevention of urolithiasis and its recurrence, even though the underlying mechanism is still unclear [6, 27]. DASH-style diet, a diet with high consumption of fruit, vegetables, and low-fat dairy products, has been reported to be associated with a decreased risk of urolithiasis [28]. In an observational study, large cohorts with adequate fluid intake, high consumption of fruits and vegetables, and low-fat dairy food, adequate calcium intake, showed a clinically meaningful reduced risk of urolithiasis [29]. However, most of the previous publications studied the effects of food intakes in combinations, and which individual food intake is beneficial for the prevention of urolithiasis is still unclear. Our MR analyses indicated that fresh fruit intake and tea intake generated a beneficial effect to prevent calculus of kidney and ureter.

One previous MR study found that tea intake was identified to reduce the risk of kidney stones [25]. The protective role of tea intake was proposed to be associated with the high content of antioxidative chemicals such as polyphenols, and caffeine that can reduce the adhesion of calcium oxalate crystals to the surface of renal tubular epithelial cells [25]. We also examined the effects of tea intake in the calculus of lower urinary tract. However, no causal relationship was identified. Our findings on fresh fruit intake are consistent with previous publications that increasing fruit intake reduced calculus risks [4, 30]. Some publications reported that the beneficial effect of fruit intake on urolithiasis is mainly mediated by the supplementation of citrate and bicarbonate [31]. Alkalosis and administration of citrate can increase urine citrate secretion and protect against stone formation [32]. A previous study on both normal and stone formers proved that withdrawing fruit and vegetable intakes for two weeks significantly reduced the urinary secretion of magnesium, citrate, potassium, and oxalate, together with an increase in urinary calcium and ammonium [33]. The European Association of Urology (EAU) guideline has encouraged the consumption of fruit and vegetables in the prevention of urolithiasis for their high content of fiber, even though the effect of vegetables are still under debate [34]. Our study may help to tailor personalized nutrition advice in the future, especially in the individuals with risk factors of urolithiasis.

There are several advantages of this current study. Firstly, the MR design we employed is suitable for causal inference. As RCT is not optional in our condition, an MR study can strengthen the causal estimation while minimizing the risk of confounding biases and reverse causality. Besides, we have performed the MR in a two-sample design, which can efficiently reduce the risk of over-fitting and false-positive findings. Additionally, we have examined the associations with several different MR methods in the sensitivity analyses, and the consistency of the results guaranteed the robustness of our findings. Furthermore, participants included in this study are constrained to the European population, which minimized the bias from population stratification. However, this also restricted the generalization of our findings to other populations. Another restriction is that using SNPs to proxy exposures mimics a life-long exposure, short-term effects of dietary habits may have a different effect. Besides, an important limitation of MR analyses is possible horizontal pleiotropy, which means that the genetic variants might affect urolithiasis not via food intakes. However, no significant pleiotropy was identified with MR-Egger analyses. Besides, the causal estimates of food intakes on urolithiasis may still be biased by potential reverse causality. For this sake, we have performed a reverse MR to estimate the effects of urolithiasis on food intakes, while no significant effects were observed. Furthermore, we employed a MVMR method to adjust the effect of fresh fruit intake on urolithiasis; however, the causal estimations may still be biased by other potential confounding factors such as lifestyle, socioeconomic status, and comorbidities. Another limitation is that the proportions of variance explained by the IVs (R^2^) are relatively low, thus null results of some associations do not necessarily mean that these food intakes are not associated with urolithiasis. Low R^2^ is also associated with a relatively low statistical power for the MR analyses. The statistical power may also be improved when future studies include a larger sample size and more cases of urolithiasis patients. Lastly, the investigation of food intakes was based on questionnaire investigations, and the accuracy of the information collection needs to be improved.

In conclusion, our MR study provided genetic evidence that fresh fruit intake may have a causal protective effect on the risk of calculus of kidney and ureter, but not lower urinary tract. An increase in the consumption of fresh fruit may provide good prevention to the development of calculus of kidney and ureter.

### Supplementary Information

**Additional file 1. **Supplementary Tables.

## Data Availability

All data generated or analyzed during this study are included in this published article and its supplementary information files. The supplementary tables can be found in: https://doi.org/10.6084/m9.figshare.22638838. Summary-level statistics from UKBB can be downloaded from MRC Integrative Epidemiology Unit (IEU) website (https://gwas.mrcieu.ac.uk/). Summary-level statistics from FinnGen Consortium can be downloaded from the FinnGen website (https://r8.finngen.fi/).
